# Allopregnanolone and Its Precursor Progesterone Do Not Reduce Injury after Experimental Stroke in Hypertensive Rats – Role of Postoperative Temperature Regulation?

**DOI:** 10.1371/journal.pone.0107752

**Published:** 2014-09-23

**Authors:** Neil J. Spratt, Amelia J. Tomkins, Debbie Pepperall, Damian D. McLeod, Mike B. Calford

**Affiliations:** 1 School of Biomedical Sciences, University of Newcastle, and Hunter Medical Research Institute, Newcastle, Australia; 2 Hunter New England Local Health District, Newcastle, Australia; 3 School of Medicine, The University of Tasmania, Hobart, Australia; Johns Hopkins University, United States of America

## Abstract

Allopregnanolone is a neurosteroid synthesized from progesterone in brain. It increases inhibition through modulation of the gamma-aminobutyric acid type A (GABA-A) receptor. Both agents are putative neuroprotectants after ischemic stroke. We sought to confirm their effectiveness in a hypertensive rat stroke model, with intra- and post-operative temperature regulation. The primary study compared allopregnanolone, progesterone or vehicle control treatments, administered 105 minutes after induction of temporary middle cerebral artery occlusion in spontaneously hypertensive rats. Temperature was controlled intraoperatively and a heat mat used in the 6 hours postoperatively to permit animal temperature self-regulation. The primary outcome was infarct volume and secondary outcomes were tests of sensory and motor function. There was no significant effect of treatment on any outcome measure. Given prior reports of GABA-A receptor agonists causing hypothermia, follow-up experiments were conducted to examine postoperative temperature regulation. These did not reveal a difference in postoperative temperature in neurosteroid-treated animals compared to control. However, in all rats maintained postoperatively in ambient temperature, moderate hypothermia was observed. This was in contrast to rats maintained over a heat mat. The lowest mean postoperative temperature was between 34.4–34.9°C in all 3 groups. These data do not support a neuroprotective effect of allopregnanolone or progesterone in ischemic stroke in hypertensives in the setting of normothermia. Given previous evidence of synergy between neuroprotective agents and hypothermia, demonstration of neuroprotective effect of these agents in the absence of postoperative hypothermia would be prudent before consideration of these agents for further clinical investigation.

## Introduction

The neurosteroid allopregnanolone is synthesized *de novo* or from systemic progesterone in neurons [1], [2]. It is the most potent known endogenous positive modulator of the inhibitory GABA-A receptor. Both allopregnanolone and its parent compound, progesterone, have been shown to reduce injury following experimental brain injury including traumatic brain injury, and stroke [3], [4], [5], [6]. Two previous studies of allopregnanolone in experimental stroke have shown a trend to greater neuroprotective efficacy than progesterone using young, healthy rats [4]. Similar or greater effect sizes have also been seen in mice. Neuroprotection was shown not to be influenced by the presence or absence of the progesterone receptor, thereby supporting the interpretation that the effect is GABA-A mediated [6]. In both of these studies, as well as in studies of other conditions, temperature was measured and regulated in the intraoperative, but not the postoperative phase. However previous investigations with another GABA-A agonist showed that this agent resulted in hypothermia postoperatively [7].

According to the recommendations of the Stroke Therapy Academic Industry Roundtable (STAIR) [8] for preclinical development of potential neuroprotectives, it is also important to confirm neuroprotection in a disease model. Therefore, the primary aim of this study was to evaluate the putative neuroprotective effect of allopregnanolone and progesterone in experimental stroke in spontaneously hypertensive rats (SHR), measuring the effect of these agents on infarct volume with secondary outcomes of neurobehavioral scores. A randomized, blinded placebo-controlled study was undertaken comparing treatment with allopregnanolone, progesterone or vehicle control after 90 minute temporary middle cerebral artery occlusion (MCAo). This methodologically rigorous design was used in accordance with good laboratory practice recommendations [9]. Particular attention was paid to body temperature. Observation of post-stroke body temperature alterations in some animals in the treatment groups led to the hypothesis that hypothermia may account for some of the neuroprotective benefits of these agents reported in previous studies, in which post-operative temperature was not recorded. A second series of experiments was therefore conducted to monitor postoperative temperature continuously with the aim of determining the effects of neurosteroids on post-stroke thermoregulation. Both aims were achieved.

## Methods

### Animals

All animal experimentation was undertaken with the approval of the animal care and ethics committee of the University of Newcastle (Approval No. 1025) and in compliance with the requirements of the Australian Code of Practice for the Care and Use of Animals for Scientific Purposes. A total of 62 male spontaneously hypertensive rats (SHR) (290–350 g) were used (Animal Resource Centre (ARC), Perth, Australia). For stroke experiments, 46 animals were used. For follow up experiments of body temperature during anesthetic recovery 16 animals were used. Animals were housed 2–3 per cage in a temperature and humidity controlled environment on a 12 hour light-dark cycle and were fed a standard laboratory rat chow.

### Surgery and administration of study compounds

MCAo was performed under isoflurane anesthesia using silicone tipped intraluminal filaments (silicone tip external diameter and length 0.35 & 2.5 mm respectively) as reported previously [10]. A simple test of forelimb flexion was performed immediately prior to re-induction of anesthesia for reperfusion at 90 minutes. Animals without evidence of neurological deficit were excluded from further study. At 105 minutes post-MCAo (while still anesthetized post-reperfusion), rats were randomized to receive 1.5 mL i.p. from a coded syringe containing allopregnanolone, progesterone (both 8 mg/kg), or vehicle (30% cyclodextran) mixed by sonication and warmed to body temperature. Six hours post-MCAo a second identical injection was given subcutaneously. Animals were only randomized to treatment following reperfusion of the MCA. Treatment was contained in identical syringes labeled with randomly generated 3 digit numbers. The allocation code was prepared by a non-randomising investigator, and kept in a sealed envelope in a locked cabinet until the completion of all analyses. Hence all investigators were blind to treatment allocation until completion of all analyses. Sample sizes were calculated to enable detection of a 30% difference between treatment groups with a power of 80% at alpha 0.05.

### Temperature regulation

During surgery, animals were maintained at 37°C on a rectal temperature regulated homeothermic heating mat (Physitemp Instruments). Heart and respiratory rates, oxygen saturations and tail-cuff systolic blood pressure (NIBP-R controller, ADInstruments) were monitored throughout surgery. Following MCAo, animals were woken from anesthetic and housed individually in cages placed ½ over a heating mat maintained at 37°C for the first 6 hours postoperatively, after which they were returned to the animal house. Rectal temperature was taken at 2, 6 and 24 hours post-stroke.

### Outcome analyses

The prespecified primary outcome was infarct volume at 24 hours, and secondary outcomes were a composite behavioral score and performance on the “sticky-dot removal test”. Neurological scoring was performed at 2 and 24 hours. This consisted of a composite score of forelimb flexion, torso twisting and lateral push [11]. At 24 hours the sticky-dot (adhesive removal) test was performed [12], [13]. In the 4 weeks prior to stroke induction, animals received at least 3 training sessions with 3 repetitions per session or until they consistently removed the adhesive label from the forepaws immediately after it was applied. For outcome analysis, speed of removal of an adhesive label from the distal forepaw was recorded for each side, and speed relative to contralateral and to pre-stroke baseline were calculated. Following final behavioral testing, animals were perfused intracardially with 4% paraformaldehyde under deep anesthesia [10]. Brains were post-fixed in 10% neutral buffered formalin, processed and paraffin embedded. Five micron thick sections at 1 mm intervals were stained with hematoxylin and eosin. Photographed areas of infarction and hemispheric areas were measured using Image J software (NIH, Bethesda). If there were any areas where there was any uncertainty regarding the presence of infarction based on the photomicrograph alone, the sections were correlated with high power microscopic evaluation of the region in question. Volumes were calculated as a percentage of the ipsilateral hemisphere.

### Effect on body temperature of recovery at ambient temperature

A secondary study was performed to investigate the possibility that allopregnanolone and/or progesterone may disrupt normal thermoregulation in the immediate postoperative phase. Animals were subjected to isoflurane anesthesia for 1 hour, during which they had a temperature datalogger (SubCue, Calgary, Canada) inserted in the extraperitoneal potential space, under the abdominal musculature. Core body temperature was logged every 5 minutes for the following 24 hours. Immediately prior to recovery from anesthesia animals received an intraperitoneal injection of allopregnanolone (n = 6), progesterone (n = 4) or vehicle (n = 6), with a follow up subcutaneous dose 6 hours later, following the same regime as for the stroke experiments, including concealment of treatment allocation. Intraoperative temperature was maintained on a homeothermic heating mat, however after waking from anesthesia animals were placed back in standard cages with bedding materials, without the use of a hot water heat mat. Ambient temperature adjacent to the cages was recorded.

Temperature data from a third cohort of animals are presented for comparison of temperature regulation after MCAo with the use of a heat mat postoperatively. These animals were normothermic control animals from a separate study of the use of hypothermia post stroke. They had stroke surgery, data logger implantation and were then placed in cages ½ over heating mats for 6 hours postoperatively, exactly as was done for the initial cohort.

### Statistics

Comparisons of infarct volumes and other continuous variables were performed using one-way ANOVA with Tukey's multiple comparisons test. Categorical variables such as behavioral scores were analysed using a Kruskal-Wallis test with post hoc Dunn's test for significant results. Temperature data in part 2 was analysed using 2-way ANOVA with repeated measures and Bonferroni post-hoc tests. Infarct volumes are presented as mean ±95% confidence intervals. Physiological and other data are presented as mean± standard deviations, unless otherwise specified.

### Exclusions

In total, 8 animals were excluded from analysis. Three had no stroke deficit prior to reperfusion, 2 had the occluding suture inadvertently completely withdrawn at reperfusion (leaving a potential silicone embolus intravascularly). Two animals were excluded due to inadvertent administration of (concealed) treatment at the incorrect time interval and 1 animal was excluded due to death at 4 hours postoperatively, rendering both infarct and behavioural outcomes unobtainable. This animal did not have a large deficit on neurological score 2 hours previously, and the death remained unexplained after post-mortem examination (this animal received allopregnanolone). A further animal (allopregnanolone) had unquantifiable infarct volume due to damage to the brain during the fixation process. Therefore for the allopregnanolone group infarct volume data n = 11, and behavioral data n = 12. Progesterone and vehicle groups had n = 13 for all data.

## Results

### Stroke Outcomes

No significant differences were detected in physiological data at baseline or during the intra- and postoperative periods between any groups (allopregnanolone, progesterone and vehicle) (Table 1).

**Table 1:** Physiological data in the three treatment groups.

	Vehicle	Progesterone	Allopregnanolone
Preoperative Weight (g)	349 (±36)	338 (±25)	357(±27)
**Intraoperative**			
Temperature (°C)	37.2(±0.4)	37.4(±0.2)	37.4(±0.3)
Heart Rate	348(±24)	342(±28)	356(±20)
Systolic Blood Pressure (mmHg)	106(±19)	124(±33)	115(±24)
Respiratory Rate	44.4(±3.8)	46.1(±4.7)	48.2(±7.0)
Oxygen Saturations (%)	97.2(±1.6)	96.5(±1.5)	95.5(±1.2)
**Reperfusion**			
Temperature (°C)	37.3(±0.6)	37.2(±0.6)	37.5(±0.9)
Heart Rate	348(±54)	356(±18)	384(±28)
Respiratory Rate	37.9(±4.7)	38.0(±6.8)	43.2(±11.0)
Oxygen Saturations (%)	96.6(±1.7)	96.4(±2.2)	97.2(±1.2)
**Postoperative**			
24 h weight loss (g)	22(±8.5)	16(±6.8)	17(±6.8)
2 h Temperature (°C)	37.3(±0.6)	37.2(±0.6)	37.3(±0.6)
6 h Temperature (°C)	37.8(±0.4)	37.5(±0.7)	37.7(±1.1)
24 h Temperature (°C)	37.2(±1.1)	36.9(±0.7)	36.6(±1.5)

Data are presented as mean ±sd.

**Figure 1:**
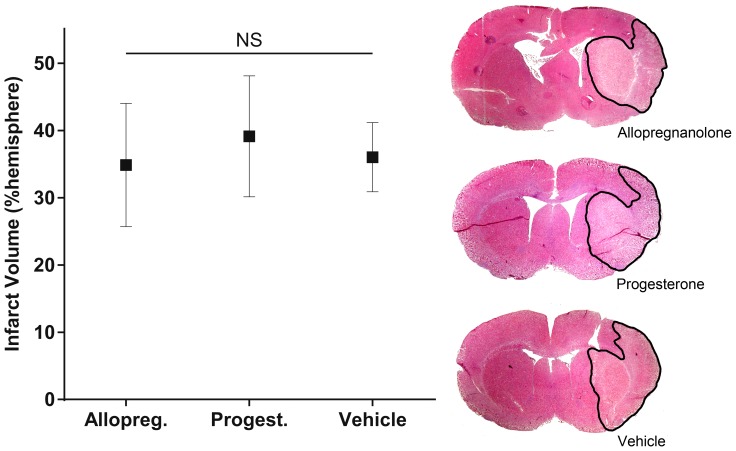
Infarct volume after administration of allopregnanolone, progesterone or vehicle control, expressed as a percentage of the ipsilateral hemisphere (mean +95% confidence intervals, allopregnanolone n = 11, progesterone n = 13, vehicle n = 13). There was no significant difference between any group. Representative H&E stained sections of each treatment group are displayed with the area of infarct outlined in black.

Infarct volumes were 34±15%, 39±15% and 36±9% (means ±95% confidence interval) of the hemispheric volume in the allopregnanolone, progesterone and vehicle treatment groups respectively (Figure. 1). There were no significant differences between groups. Similarly, there were no significant differences between groups on any individual or composite behavioral scores (Table 2). Despite no significant differences at any time point of the group means of postoperative temperature, at 24 hours there were three outlier animals with core temperature below 35°C. This was the only time point at which cages were not ½ over a heat mat, and all the outliers were from the allopregnanolone group. This observation prompted the second series of experiments to evaluate body temperature changes during the early post-operative recovery period.

**Table 2:** 24 hour behavioral score and sticky dot removal test scores.

Test	Allopregnanolone	Progesterone	Vehicle	P
Forelimb flexion	0.40±0.32	0.92± 0.52	1.23± 0.39	0.06
Torso twisting	0.60± 0.32	0.69± 0.47	0.77± 0.39	0.90
Lateral push	0.60± 0.32	0.31± 0.26	0.54± 0.28	0.33
Total	1.60± 0.67	1.92± 0.98	2.62± 0.79	0.23
Sticky dot removal (s)	283± 182	138± 117	174± 31	0.35

### Postoperative temperature changes

**Figure 2:**
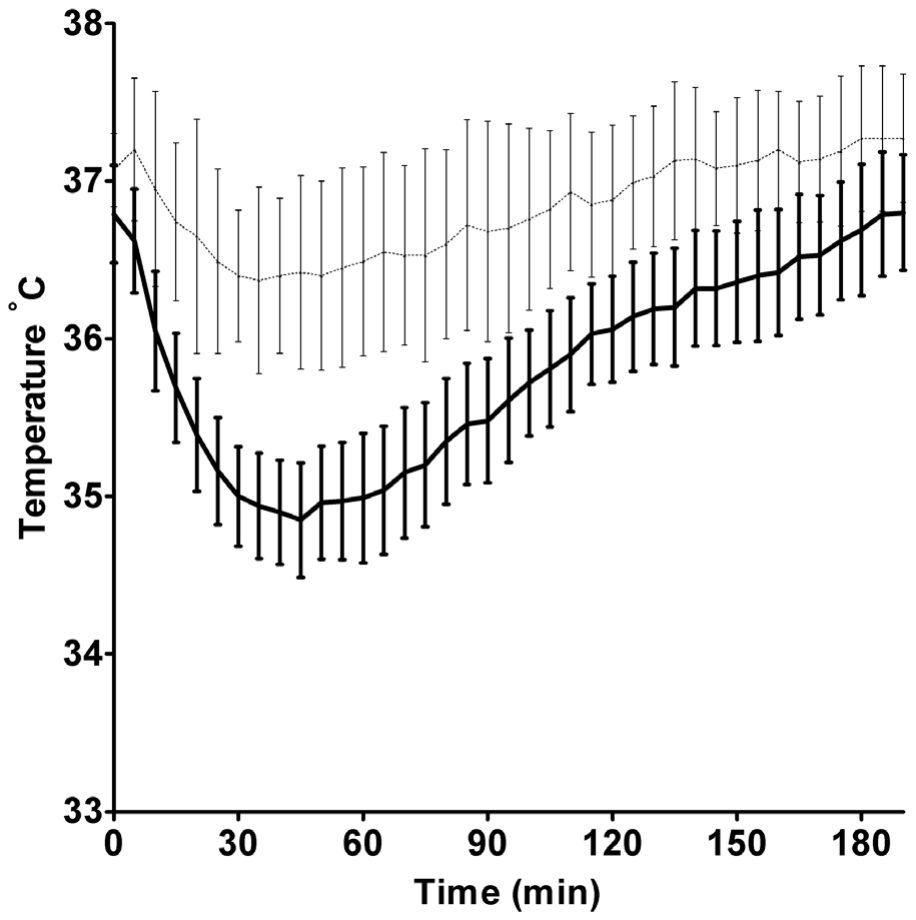
Core body temperatures during the first 3 hours of recovery following a 1 hour anesthetic. Data are presented as mean ±95% C.I. Animal temperatures were recorded by extraperitoneal datalogger. Dark lines are pooled data from the 3 treatment groups (non-stroke surgery animals, n = 16) that recovered from anesthesia in regular cages at ambient laboratory temperature (21.2±0.5°C). There was no statistically significant difference in postoperative temperatures between these 3 groups. For comparison, lighter lines are data from 9 animals that were recovered in cages ½ over a heat mat. These animals formed the normothermic control group for a separate study of hypothermia, and received stroke surgery +3 hours anesthesia before recovery. Temperatures in both groups were regulated during anesthesia. There was a statistically significant difference between animals warmed postoperatively to those kept at room temperature (p<0.0001).

In the second series of experiments (body temperature during recovery), there were no significant differences in ambient air temperature adjacent to the recovery cages. Ambient temperatures were 21.2±0.3, 21.3±0.7 and 21.1±0.4°C next to the allopregnanolone, progesterone and vehicle group cages, respectively. In all animals, core body temperatures dropped by 5–7% of baseline to, 34.9±0.4°C, 34.4±0.8°C, and 34.4±0.4°C for allopregnanolone, progesterone and vehicle groups respectively. These low temperatures occurred at a mean time of 44±20 minutes, 45±14 minutes, and 48±10 minutes post recovery from anesthesia for allopregnanolone, progesterone and vehicle groups respectively. There were no significant temperature differences between treatment groups at any time. The mean temperatures of all groups at each time point are presented at Figure. 2 (note these are somewhat higher than the numbers presented above, due to pooling of groups and the resultant variability in timing of the lowest temperature in each animal). Datalogger insertion had no significant effect on body temperature, since the mean rectal temperatures obtained in the initial experiments (stroke + treatment) at 2, 6 and 24 hours were not significantly different to the datalogger temperatures at the same times in the separate post-stroke comparison group. Temperatures at 2 h were 37.3±0.6 and 36.9±0.6°C, at 6 h were 37.6±0.7 and 37.7±0.3°C, and at 24 h were 36.9±1.1 and 37.5±0.6°C for rectal and datalogger temperatures, respectively.

## Discussion

We found that allopregnanolone and progesterone had no neuroprotective effect in hypertensive animals when body temperature was carefully maintained during and after surgery (Figure 1). Neither drug had an independent effect on body temperature (Figure 2). These randomized, blinded and appropriately powered data suggest that neither drug is likely to be of major benefit to hypertensive patients, which comprise >50% of the stroke population.

This data is in stark contrast to previously published studies of the use of allopregnanolone in experimental stroke in rats and mice [4], [6]. The dosage of the steroids used in the present study match those found effective in the earlier studies. Results are also in contrast to multiple studies of the use of progesterone in experimental stroke [4], [14], [15], [16], [17], [18], [19], as well as the use of both agents in other models of brain injury [3], [5], [20], [21], [22], [23], [24], [25], [26], [27], [28]. However, there have been other similarly negative studies in ovariectomised female rats treated with either allopregnanolone or progesterone [29], [30]. Additionally, a recent single animal data meta-analysis of progesterone neuroprotection after stroke presented evidence of significant publication bias – i.e. that negative studies may not have been published [29]. What might account for the difference of the current study from those showing neuroprotection?

One significant difference between this study and previous neuroprotection studies using these agents (in stroke and other models of neural injury), is the provision of warming during the postoperative period (6 hours), and the measurement of body temperature during this interval. All mammals including humans are prone to development of postoperative hypothermia if warming is not provided [31]. This can be profoundly neuroprotective and has led to false positive detection of neuroprotection, a phenomenon reported for other GABAergic agents such as clomethiazole [7]. Neither allopregnanolone nor progesterone altered body temperature beyond the moderate hypothermia induced by anesthesia for several hours in all non-stroked animals (Figure 2). However, some other neuroprotective agents have been shown to have a synergistic effect with mild hypothermia [32], [33]. Such synergy could be exploited for treatment, however inducing even mild hypothermia in patients is far more difficult than in postoperative small animals.

Another important difference was the use of spontaneously hypertensive rats. Few stroke neuroprotection studies examine efficacy in the face of the co-morbidities common in patients, however this was a key recommendation of the STAIR committee update [34]. While the benefits of hypothermia are maintained in the face of hypertension [35], this does not appear to always be the case for neuroprotective agents in experimental models. The degree of neuroprotection produced by the spin-trap agent NXY-059 in SHRs was significantly less than that in normotensive strains [36]. This agent was not shown to have any benefit in the subsequent large-scale clinical trial [37]. While neuroprotection by progesterone has been reported after MCAo in SHRs [18], those experiments were not temperature controlled in the postoperative phase.

Timing of stroke treatment with neuroprotectants is also critical. Reports on the importance of reperfusion injury [38] also suggest that treatment administration prior to reperfusion [4], rather than after, may be important in the neuroprotective activity of these agents. However, such a hypothesis is not consistent with reported neuroprotection by progesterone following permanent MCAo [19].

Potential limitations of this study are that we used an early sacrifice time (24 h) and that we only investigated one dose of the neurosteroids. Had there been benefit of treatment at 24 h, confirmation of neuroprotection would have been required with longer survival studies to exclude the possibility of treatment merely delaying rather than preventing infarct progression The dose of progesterone used was that previously found effective in both SHR and other strains [18], and the allopregnanolone dose chosen was based on that shown to be successful in other strains of rat in studies of occlusion and neurotrauma [4], [27], [28]. The lack of treatment effect in this study may suggest a need for further dose studies to determine the full limitations of neuroprotection of these neurosteroids, and whether higher doses are effective in hypertensives.

This study was designed in order to avoid some of the experimental factors that have been identified as being of importance in previous failures of translation of successful neuroprotectants from the laboratory into clinical practice [36]. The use of *a priori* sample size calculations, randomization, concealment of treatment allocation and blinded outcome assessment have been shown to be important aspects of trial design, preventing overestimate of efficacy of the intervention [9], [39], [40]. An important corollary is that implementation of such good laboratory practice is expected to reduce the number of ‘false positive’ results, and will therefore increase the number of negative results.

In contrast to prior studies, the current data demonstrate no difference in infarct volume or functional outcome measures compared to vehicle control in animals receiving either allopregnanolone or progesterone at standard doses. The use of postoperative temperature maintenance in this study, in contrast to previous investigations, raises the possibility that these agents are not effective at normal body temperatures or in the setting of hypertension – both of which are most commonly present in stroke patients. These findings suggest a need for further experimental study to determine the suitability of allopregnanolone or its precursors for clinical trial.
